# Atherogenic index of plasma and coronary artery disease: A systematic review

**DOI:** 10.1515/med-2022-0590

**Published:** 2022-12-06

**Authors:** Juan R. Ulloque-Badaracco, Enrique A. Hernandez-Bustamante, Esteban A. Alarcon-Braga, Melany D. Mosquera-Rojas, Alvaro Campos-Aspajo, Farley E. Salazar-Valdivia, Valeria A. Valdez-Cornejo, Vicente A. Benites-Zapata, Percy Herrera-Añazco, Germán Valenzuela-Rodríguez, Adrian V. Hernandez

**Affiliations:** Escuela de Medicina, Universidad Peruana de Ciencias Aplicadas, Lima, Perú; Sociedad Científica de Estudiantes de Medicina de la Universidad Peruana de Ciencias Aplicadas, Lima, Perú; Sociedad Científica de Estudiantes de Medicina de la Universidad Nacional de Trujillo, Trujillo, Perú; Grupo Peruano de Investigación Epidemiológica, Unidad Para la Generación y Síntesis de Evidencias en Salud, Universidad San Ignacio de Loyola, Lima, Perú; Unidad de Investigación para la Generación y Síntesis de Evidencias en Salud, Vicerrectorado de Investigación, Universidad San Ignacio de Loyola, Avenida La Fontana #750 La Molina, Lima, Perú; Facultad de Ciencias de la Salud, Universidad Privada del Norte, Trujillo, Perú; Instituto de Evaluación de Tecnologías en Salud e Investigación – IETSI, EsSalud, Lima, Peru; Clínica Delgado, Servicio de Medicina Interna y Cardiología, Lima, Perú; Unidad de Revisiones Sistemáticas y Meta-análisis (URSIGET), Vicerrectorado de Investigación, Universidad San Ignacio de Loyola (USIL), Lima, Perú; Unidad de Revisiones Sistemáticas y Meta- análisis, Guías de Práctica Clínica y Evaluaciones de Tecnología Sanitaria, Vicerrectorado de Investigación, Universidad San Ignacio de Loyola, Lima, Perú; Health Outcomes, Policy, and Evidence Synthesis (HOPES) Group, University of Connecticut School of Pharmacy, Storrs, CT, United States of America

**Keywords:** coronary disease, dyslipidemias, atherosclerosis, lipids, heart diseases

## Abstract

Various studies suggest that the atherogenic index of plasma (AIP) is associated with the risk of coronary artery disease (CAD) in different clinical scenarios. This review aimed to synthesize evidence of the association between AIP values and CAD. A literature search was carried out on four databases, namely, PubMed, Scopus, Web of Science, and Ovid-Medline. A handsearch was performed on preprint repositories (MedRxiv and Research Square). The effect measurements were expressed as odds ratios (OR) with their corresponding 95% confidence intervals (CI). For the quantitative synthesis, we employed a random-effects model. We analyzed 14 articles (with 40,902 participants) from seven different countries. The quantitative analysis revealed that an increase in one unit of AIP was associated with higher odds of developing CAD (OR 2.11; 95% CI 1.65–2.69; *P* < 0.001; *I* ^2^ = 98%). We conducted subgroup analyses of Chinese (OR 1.89; 95% CI 1.40–2.56; *P* < 0.001) and non-Chinese studies (OR 2.51; 95% CI 1.42–4.42; *P* < 0.001). The sensitivity analysis by risk of bias continued to demonstrate an association, and the heterogeneity remained unchanged (OR 1.75; 95% CI 1.33–2.31; *P* < 0.001; *I* ^2^ = 98%). Higher AIP values were associated with higher odds of developing CAD.

## 1 Introduction

Coronary artery disease (CAD), also referred to as coronary heart disease (CHD) or ischemic heart disease, is a disease with a high prevalence and is one of the leading causes of death worldwide [1,2,3]. In the last few decades, as preventive and treatment measures have strengthened and improved, the incidence and mortality of this pathology have decreased in developed countries [4,5,6,7]. However, the global prevalence is expected to increase to 1,845 cases per 100,000 population by 2030 [1].

Cardiac risk stratification is necessary to improve preventive and therapeutic interventions [8]. Biomarkers are an essential component in this task, either as part of prediction models or on their own. In recent years, multiple CAD risk biomarkers have been identified and investigated, including C-reactive protein [9], lipoprotein-associated phospholipase A2 [10], and fibrinogen [11], among others. The atherogenic index of plasma (AIP) is a novel biomarker consisting of the logarithmically transformed ratio of triglycerides to high-density lipoprotein (HDL)-cholesterol in molar concentrations. Unlike biomarkers such as serum amyloid alpha [12] or matrix metalloproteinases [13], AIP is an easily accessible biomarker because only a standard lipid profile is required for its calculation [14].

Hypertriglyceridemia and low HDL levels are independent markers of CHD [15,16]. Therefore, the AIP combines these two lipid parameters into a single biomarker to boost its predictive capabilities.

Thus, several studies have suggested that AIP acts as a marker associated with cardiovascular diseases in patients with various pathologies [17,18,19,20,21,22]. This association is related to a surrogate involvement of endothelial vascular damage, such as carotid intima-media thickening, compromised atrial coronary anatomical, or clinical conditions, e.g., stroke and acute coronary syndromes, as a manifestation of CAD. However, to the best of our knowledge, the evidence of the association between AIP and CAD has not been systematized. Thus, this review aimed to synthesize such evidence.

## 2 Methods

### 2.1 Study design, registration, and report guideline

We conducted this systematic review in accordance with the guidelines of the Preferred Reporting Items for Systematic Reviews and Meta-Analysis (PRISMA) statement [23]. The summarized version of the protocol was registered in the International Prospective Register of Systematic Reviews with code CRD42021289308 (see PRISMA checklist in Table S1).

### 2.2 Data sources and searches

We conducted a literature search on articles that evaluated the association between AIP and CAD on October 15, 2021. Four databases were searched: PubMed, Scopus, Web of Science, and Ovid-Medline. A handsearch was also performed on preprint repositories (MedRxiv and Research Square) (see Supplementary Appendix 1).

### 2.3 Study selection and data extraction

The articles collected from the aforementioned databases were exported to the Rayyan data management software [24], and duplicate studies were eliminated. Four reviewers independently screened each study (A.C.-A., E.A.A.-B., E.A.H.-B., and J.R.U.-B.) by analyzing the titles and abstracts according to the selection criteria. We included (1) case–control and cohort studies that (2) evaluated the association between AIP and CAD in (3) adults (≥18 years old). We excluded articles that were (1) case reports, (2) studies conducted on animals, (3) scoping reviews, (4) narrative reviews, and (5) systematic reviews.

Once this phase was completed, each of the articles was read in full-text, and we determined whether they met the previously established selection criteria. A study was excluded if it failed to comply with the selection criteria. If an article did not contain the required information, an attempt was made to contact the corresponding author of the said article. A secondary bibliographic search was conducted on the articles that reached the full-text review phase.

In case of disagreements on the inclusion or exclusion of an article, a third reviewer was consulted. Finally, information of the selected articles was extracted and collated in a standardized Excel 2016 document. The extracted data included author name, country, year of publication, study sample, study design, population characteristics (age, gender, etc.), exposure (AIP mean or median of the whole sample and according to sample stratification), and crude/adjusted association measures (odds ratio [OR], relative risk [RR], and hazard ratio). Outcome definitions are presented in Table S2.

### 2.4 Assessment of study quality and publication bias

Four authors (A.C.-A., V.A.V.-C., M.D.M.-R., and F.E.S.-V.) evaluated the included studies independently using the Newcastle–Ottawa Scale [24]. The studies were stratified into three levels of methodological quality according to the number of stars: low risk of bias (≥6 stars), moderate risk of bias (4–5 stars), and high risk of bias (<4 stars). In addition, funnel plots and Begg’s test were conducted to assess publication bias; *P*-values > 0.1 were considered indicative of no publication bias.

### 2.5 Data synthesis and analysis

The quantitative analysis was conducted using the RevMan 5.4 statistical program (Cochrane Collaboration, Copenhagen, Denmark). The OR and their corresponding 95% confidence intervals (CI) were the only effect measures used; thus, we transformed standardized mean differences to ln[OR] using the Chinn method. RR was converted into OR [25,26]. Variables expressed as median and interquartile range were converted to means and standard deviation using Hozo’s method [27]. We assessed statistical heterogeneity by calculating the *I* ^2^-statistic, where values >60% were considered to indicate severe heterogeneity. A Cochran’s *Q* test was also conducted, where a *P*-value < 0.05 was considered statistically significant. A random-effects model was used for the meta-analysis as we anticipated heterogeneity among studies. In the subgroup analysis, we conducted a comparison between associations according to country (Chinese vs non-Chinese studies), study design, CAD definition, and gender (women vs mixed). In the sensitivity analysis, we excluded studies with a high risk of bias. We also analyzed all studies after transforming OR to RR. In addition, we performed a meta-regression, reported as bubble plots, in order to search for heterogeneity sources, due to differences in the clinical and methodological characteristics of the individual studies. Assessed variables were study design, study location, CAD definition, and gender.

## 3 Results

### 3.1 Study selection

**Figure 1:**
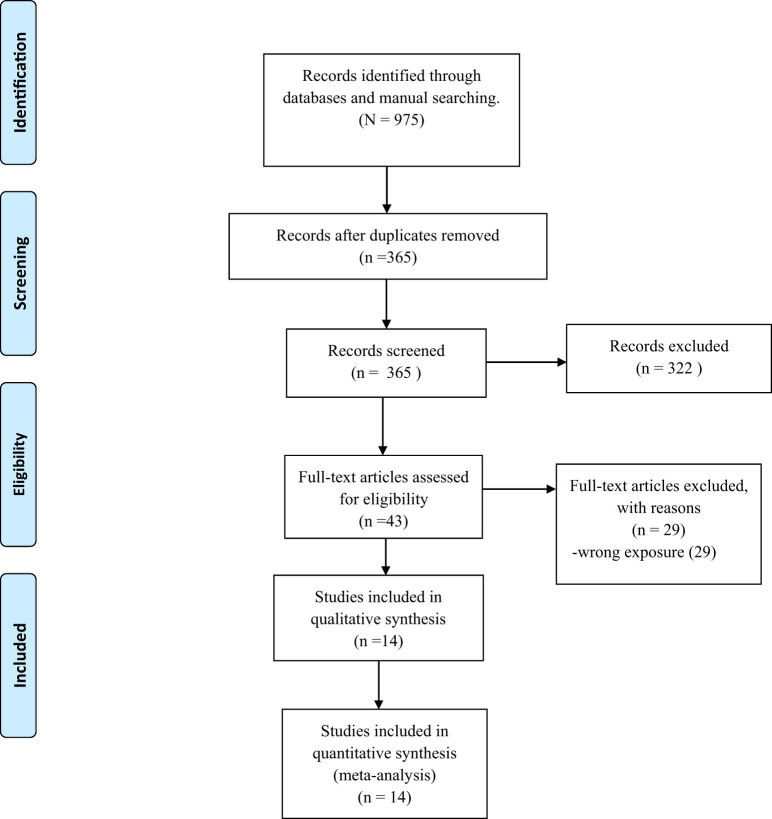
PRISMA flow diagram.

The search strategy yielded 975 records. After removing 610 duplicates, 365 articles finally remained. After evaluating the titles and abstracts, we excluded 322 more studies. Then, a full-text review was performed on the remaining 43 articles, which led to the exclusion of 29 more articles. In the end, 14 articles were included in the qualitative analysis and meta-analysis [20,28,29,30,31,32,33,34,35,36,37,38,39,40]. This process is documented in a PRISMA flow diagram (Figure 1).

### 3.2 Study characteristics

The characteristics of each study are presented in detail in Table 1. Most of the articles (8 out of 14) were case–control studies, and six were cohort studies. The distribution of the studies by country was as follows: China (7), India (1), Turkey (2), Egypt (1), South Korea (1), Slovakia (1), and the USA (1). In total, there were 40,902 participants with samples ranging from 80 to 11,164 participants. The gender distribution was as follows: 17,026 were male, and 23,876 were female. The study by Celik et al. reported an optimal cutoff value of AIP for CAD of 0.541 and an area under the curve of 0.617 [30].

**Table 1:** Clinical characteristics of the included studies

Author	Year	Country	Participants (male/female)	Median/mean age (Interquartile range\standard deviation (SD))	Study design	AIP mean (SD) in CAD patients	AIP mean (SD) in non-CAD patients	SMD between CAD and non-CAD patients	OR (95% CI), *P*-value (crude)	OR (95% CI), *P*-value (adjusted)
Onat et al.	2010	Turkey	2,676 (1,294/1,392)	49 (13)	Cohort	NR	NR	NR	NR	1.34 (1.11–1.61), *P* < 0.001^a^
Raj-Sedhai et al.	2018	USA	313 (169/144)	63 (12)	Cohort	0.39 (0.03)	0.35 (0.02)	1.51 [1.25, 1.76]	NR	NR
Al-Shaer et al.	2021	Egypt	140 (NR/NR)	NR	Case–control	0.20 (0.27)	0.1 (0.27)	0.37 [0.03, 0.70]	NR	NR
Celik et al.	2019	Turkey	280 (154/126)	64 (10)	Case–control	0.62 (0.30)	0.48 (0.28)	0.48 [0.24, 0.72]	NR	NR
Wang et al.	2021	China	3,600 (2,243/1,357)	60 (10)	Cohort	NR	NR	NR	2.6 (2.381–2.8348), *P* < 0.001	NR
Won et al.	2020	South Korea	6,928 (3,977/2,951)	52 (10)	Cohort	NR	NR	NR	1.1 (1.07–1.13), *P* < 0.001	NR
Guo et al.	2020	China	4,644 (0/4,644)	64 (8)	Case–control	0.15 (0.28)	0.10 (0.28)	0.18 [0.12, 0.24]	NR	NR
Wu et al.	2018	China	696 (0/696)	61 (7)	Case–control	0.20 (0.27)	0.10 (0.27)	0.37 [0.03, 0.70]	NR	NR
Cai et al.	2017	China	5,387 (3,242/2,145)	62 (9)	Case–control	0.17 (0.30)	0.12 (0.31)	0.16 [0.11, 0.22]	NR	NR
Pridavkova et al.	2015	Slovakia	80 (43/37)	NR	Cohort	0.22 (0.29)	0.17 (0.34)	0.16 [−0.29, 0.61]	NR	NR
Shanker et al.	2016	India	11,164 (7,379/3,515)	53 (8)	Cohort	NR	NR	NR	1.80 (1.54–2.1), *P* < 0.001	NR
Dai et al.	2012	China	238 (0/238)	65 (7)	Case–control	0.61 (0.14)	0.53 (0.09)	0.67 [0.41, 0.93]	NR	NR
Zhou et al.	2021	China	3,278 (2,473/805)	59 (10)	Case–control	0.37 (0.23)	0.25 (0.26)	0.50 [0.42, 0.57]	NR	NR
Gaojun et al.	2019	China	1,478 (1,373/1,05)	33 (30–35)	Case–control	0.35 (0.30)	0.21 (0.33)	0.45 [0.34, 0.57]	NR	NR

a: Adjusted to age and serum C-reactive protein. NR: Not reported.

### 3.3 Assessment of study quality and publication bias

Table S3 presents the results of the methodological quality assessment. In summary, 10 and 4 studies were classified as having a low and moderate risk of bias, respectively.

### 3.4 AIP and risk of CAD

**Figure 2:**
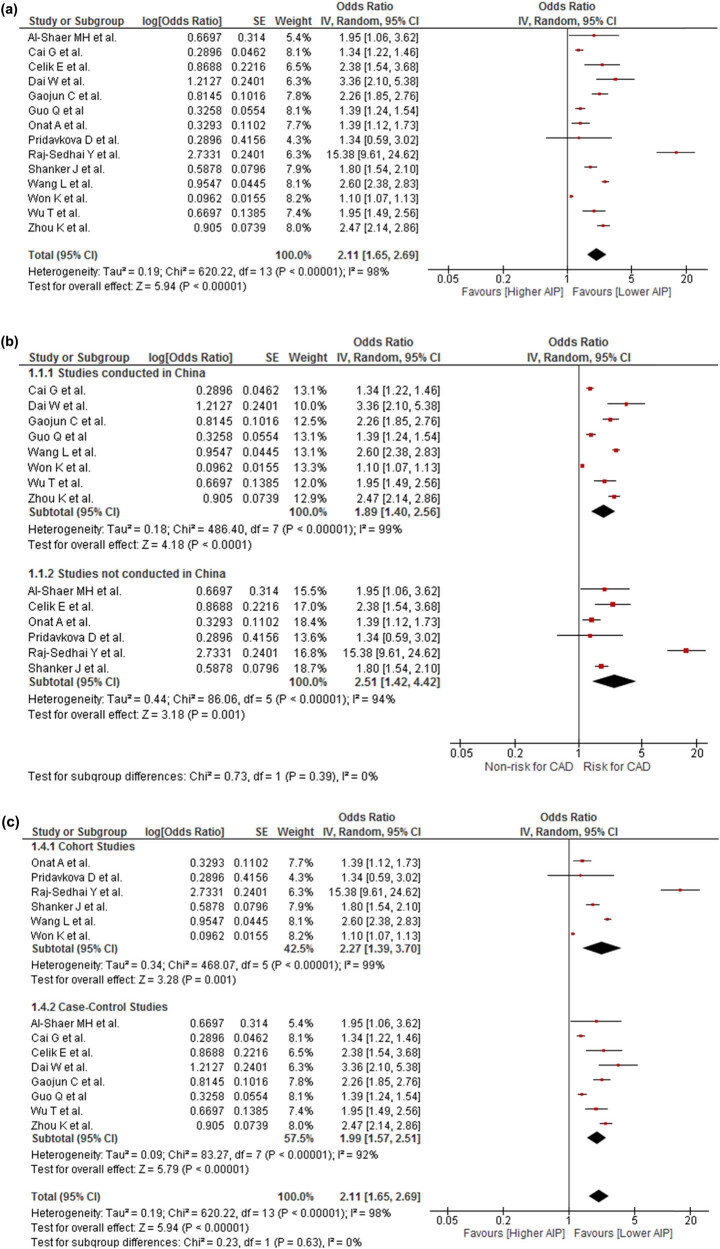

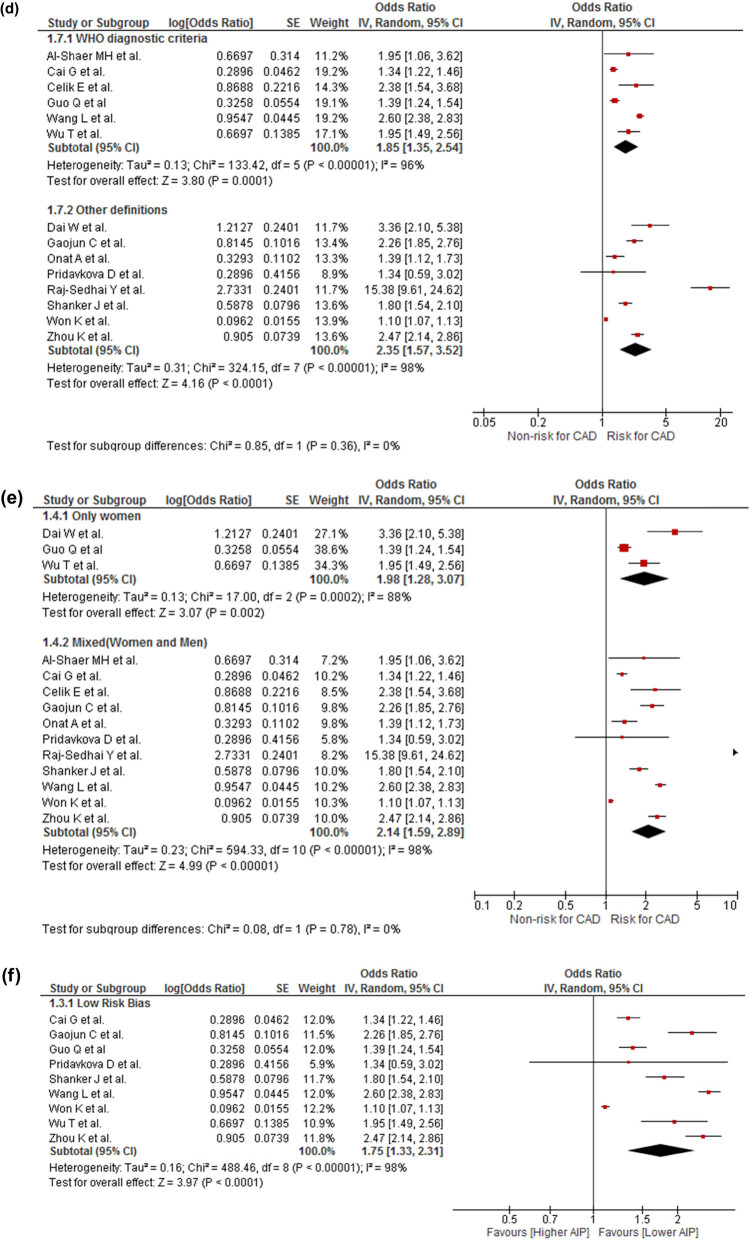

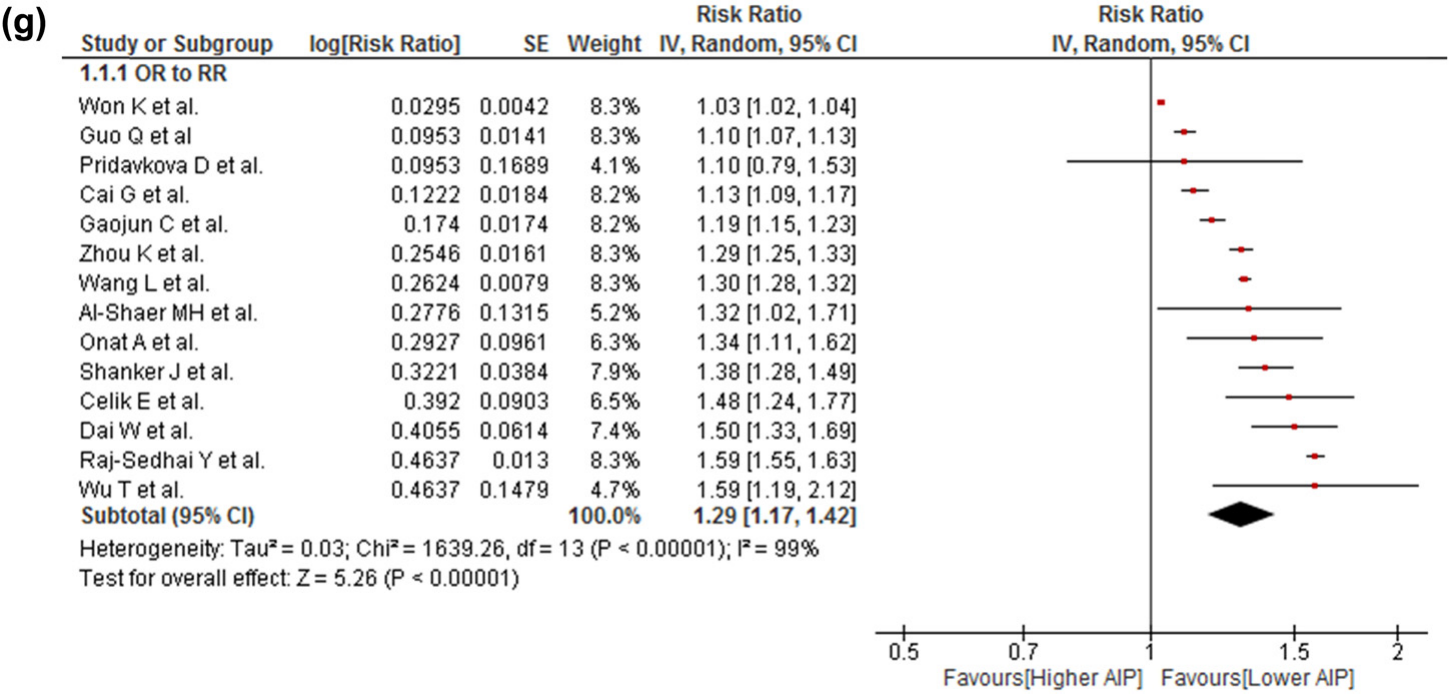
(a) Association of AIP and risk for CAD. (b) Subgroup analysis according to the origin country of the association between AIP and risk for CAD. (c) Subgroup analysis according to the study design of the association between AIP and risk for CAD. (d) Subgroup analysis according to CAD definition of the association between AIP and risk for CAD. (e) Subgroup analysis according to the gender of the association between AIP and risk for CAD. (f) Sensitivity analysis according to the risk of bias of the association between AIP and risk for CAD. (g) Sensitivity analysis according to the transformation from OR to RR.

The quantitative analysis revealed that patients with higher AIP values were associated with higher odds of developing CAD (OR 2.11; 95% CI 1.65–2.69; *P* < 0.001) (Figure 2a) with severe heterogeneity (*I* ^2^ = 98%). Statistically significant associations with high heterogeneity were observed in all subgroup analyses by study location (Figure 2b), study design (Figure 2c), definition of CAD (Figure 2d), and gender (Figure 2e). Sensitivity analyses were conducted. In one analysis, only studies with a low risk of bias (Figure 2f) were included (OR 1.75; CI 95% 1.33–2.31; *P* < 0.001; *I* ^2^ = 98%), whereas in the other, all OR values were transformed to RR (RR 1.29; CI 95% 1.17–1.42; *P* < 0.001; *I* ^2^ = 99%); however, the association with high heterogeneity remained in both cases (Figure 2g).

### 3.5 Meta-regression

No significantly associated coefficient was observed in study location (*P* = 0.974) (Figure S1), study design (*P* = 0.959) (Figure S2), CAD definition (*P* = 0.614) (Figure S3), or gender (*P* = 0.958) (Figure S4).

### 3.6 Publication bias

There was no indication of small-study effects (Begg’s test, *P* = 0.956), and the funnel plots did not show asymmetry (Figure S5).

## 4 Discussion

From our systematic review and meta-analysis, we obtained sufficient evidence to consider AIP as a marker of CAD risk in the general population.

Multiple studies suggested the value of AIP as a predictor of fatty liver [41], hidradenitis suppurativa [42], osteomyelitis in patients with diabetic foot [43], obesity [44], diabetes [45], and an estimate-reduced glomerular filtration rate [46]. These associations are related to oxitdative stress [47], insulin resistance and hyperinsulinemia [45], and mechanisms associated with atherosclerosis [43,44], which are common risk factors for cardiovascular disease.

AIP is positively correlated with the fractional esterification rate of HDL, especially with small dense low-density lipoprotein (sdLDL), whose expression in AIP reflects the complex interactions of lipoprotein metabolism and is useful in predicting plasma atherogenicity [48]. sdLDL has atherogenic properties, such as reduced clearance from the bloodstream, enhanced oxidation compared with other lipoproteins, enhanced binding to endothelial proteoglycans, enhanced penetration across the endothelial barrier, and increased uptake by macrophage receptors [49]. These properties increase the formation of foam cells and are the initial stages of atherogenesis [50]. These factors could explain the results of different studies indicating an association between AIP and CAD [51,52,53], which varies depending on the value of this marker. In this way, Dobiásová suggested that AIP values between −0.3 and 0.1 were associated with low cardiovascular risk and AIP values from 0.1 to more than 0.24 were associated with high cardiovascular risk [14].

For the aforementioned reasons, our finding of an association between high AIP values and increased CAD risk is not surprising. In addition to the aforementioned factors, factors associated with specific CAD conditions could also explain our results. In this context, some studies have found an association between AIP and arterial stiffness in normotensive patients [54], which is a risk factor for CAD [55]. Choudhary et al., in a study conducted on subjects without antihypertensive or lipid-lowering therapy, found that AIP was related to pulse wave velocity but not to aortic or radial blood pressure, cardiac output, or systemic vascular resistance [54] Similarly, other studies associated AIP with the progression of coronary calcification, a known surrogate marker of CAD [56]. Two studies from Korea found a significant correlation between AIP and coronary calcification progression in subjects without cardiovascular disease [57], although AIP was not an independent predictor [57].

Contrarily, in a multinational study in seven countries from 2003 to 2015, this association was independent of traditional risk factors [58]. Similarly, AIP was also a predictor of subclinical CAD. A study of patients with or without chronic kidney disease found that AIP was associated with advanced subclinical CAD after adjustment for age older than 60 years, male sex, hypertension, diabetes mellitus, dyslipidemia, obesity, and proteinuria [59].

Finally, some studies demonstrated an association between AIP and the evolution of patients with acute coronary syndromes and myocardial infarction. For example, a Turkish study found that AIP was independently associated with the absence of reflow in patients with ST-elevated myocardial infarction following the primary percutaneous coronary intervention [52]. Surprisingly, a study in Jakarta found that patients hospitalized following myocardial infarction with AIP values <0.24 had almost four times higher mortality than those who did not have [60]. Although the authors did not find an explanation for their results, they suggested the role of the patients’ diet as a possible reason.

The meta-analysis conducted by Wu et al. [61] found that high AIP values are associated with an increased risk of AIP; however, this meta-analysis has many inconsistencies, such as the incorrect grouping of values in the overall meta-analysis, as they placed AIP values according to gender, which should have been done in a subgroup analysis according to the participants’ gender. Another error in this manuscript is the lack of explanation of how the AIP values were obtained for the subgroup analysis according to the patients’ health status because there is no such information in the primary studies they used.

Despite the evidence suggesting an association between AIP and CAD, this is a systematic review and meta-analysis that systematize the available evidence. We also conducted sensitivity analyses considering the biases of the studies and the place of origin of the studies, which provided robustness to our results. The findings obtained allow us to suggest the use of AIP as a potential low-cost marker of CAD that will allow health personnel to prioritize management strategies and could even be used as part of predictive scores, replacing markers such as triglycerides. Similarly, the usefulness of this marker is independent of the age of the patients [39,62], although, concerning gender, some studies suggested that, at least in young patients, its predictive value is mainly in males [39], which should be verified in subsequent studies. However, despite the fact that AIP is a marker that is calculated using parameters that are easy to obtain in clinical practice and that our results indicate its usefulness, the studies included in our systematic review are flawed and have small sample sizes. In this sense, despite being promising, more studies with better design are needed before adopting it in usual clinical practice.

Our systematic review has some limitations that must be acknowledged. First, there is evidence of high statistical heterogeneity due to the clinical and methodological differences between the analyzed studies. Second, most studies have not controlled the AIP values for confounding variables that impact the proposed outcome. Sociodemographic lifestyles and comorbidity variables should be considered in future studies to avoid confounding bias. Despite this, the direction of the effect is consistent in most of the reported studies. Third, most studies were conducted in Asia, with few or no studies found in other continents. Therefore, it is important to analyze the AIP value in CAD risk in these populations to establish its usefulness. Fourth, no definitive cutoff points have been reported to set the sensitivity and specificity of the AIP in CAD; thus, it is necessary to address it in future research in various contexts and populations. Finally, our findings indicate that high AIP values are associated with a greater possibility of suffering from CAD; however, with the obtained evidence, we consider that AIP is a risk marker and not a causal risk factor because the causes of lipid alterations are mediated by genetic and environmental factors. The AIP in altered ranges would be the expression of this multicausality and, therefore, a biomarker of the pathophysiological process leading to the appearance of CAD.

## 5 Conclusion

This systematic review and meta-analysis demonstrated that patients with higher AIP values have higher odds of developing CAD. However, additional primary studies are warranted to define an optimal cutoff point of AIP to predict CAD.

## Supplementary Material

Supplementary Material

## References

[1] Khan MA, Hashim MJ, Mustafa H, Baniyas MY, Suwaidi SKBM, Al AlKatheeri R, et al. Global epidemiology of ischemic heart disease: results from the global burden of disease study. Cureus. 2020;12:e9349. 10.7759/CUREUS.9349. PMC7384703 32742886

[2] Virani SS, Alonso CA, Aparicio HJ, Benjamin EJ, Bittencourt MS, Callaway CW, et al. Heart disease and stroke statistics – 2021 update a report from the American Heart Association. Circulation. 2021;143:e254–743. 10.1161/CIR.0000000000000950. PMC13036842 33501848

[3] Virani SS, Alonso A, Benjamin EJ, Bittencourt MS, Callaway CW, Carson AP, et al. Heart disease and stroke statistics – 2020 update: a report from the American Heart Association. Circulation. 2020;141:e139–596. 10.1161/CIR.0000000000000757. 31992061

[4] Sanchis-Gomar F, Perez-Quilis C, Leischik R, Lucia A. Epidemiology of coronary heart disease and acute coronary syndrome. Ann Transl Med. 2016;4:256. 10.21037/ATM.2016.06.33. PMC4958723 27500157

[5] Luepker RV. Falling coronary heart disease rates: a better explanation. Circulation. 2016;133:8–11. 10.1161/CIRCULATIONAHA.115.019862. PMC4768868 26582782

[6] Bhatnagar P, Wickramasinghe K, Wilkins E, Townsend N. Trends in the epidemiology of cardiovascular disease in the UK. Heart. 2016;102:1945–52. 10.1136/HEARTJNL-2016-309573. PMC5256396 27550425

[7] Mensah GA, Wei GS, Sorlie PD, Fine LJ, Rosenberg Y, Kaufmann PG, et al. Decline in cardiovascular mortality: possible causes and implications. Circulation Res. 2017;120:366–80. 10.1161/CIRCRESAHA.116.309115. PMC5268076 28104770

[8] Kullo IJ. Novel biomarkers of coronary artery disease risk. Mayo clinic cardiology: concise textbook. 4th ed. New York: Oxford University Press; 2013. 10.1093/MED/9780199915712.003.1166.

[9] Casas JP, Shah T, Hingorani AD, Danesh J, Pepys MB. C-reactive protein and coronary heart disease: a critical review. J Intern Med. 2008;264:295–314. 10.1111/J.1365-2796.2008.02015.X. 18823504

[10] Khuseyinova N, Imhof A, Rothenbacher D, Trischler G, Kuelb S, Scharnagl H, et al. Association between Lp-PLA2 and coronary artery disease: focus on its relationship with lipoproteins and markers of inflammation and hemostasis. Atherosclerosis. 2005;182:181–8. 10.1016/J.ATHEROSCLEROSIS.2004.10.046. 16115490

[11] Morange PE, Bickel C, Nicaud V, Schnabel R, Rupprecht HJ, Peetz D, et al. Haemostatic factors and the risk of cardiovascular death in patients with coronary artery disease: the AtheroGene study. Arteriosclerosis Thrombosis, Vasc Biol. 2006;26:2793–9. 10.1161/01.ATV.0000249406.92992.0d. 17023678

[12] Johnson BD, Kip KE, Marroquin OC, Ridker PM, Kelsey SF, Shaw LJ, et al. Serum amyloid A as a predictor of coronary artery disease and cardiovascular outcome in women: the National Heart, Lung, and Blood Institute-Sponsored Women’s Ischemia Syndrome Evaluation (WISE). Circulation. 2004;109:726–32. 10.1161/01.CIR.0000115516.54550.B1. 14970107

[13] Hassanzadeh-Makoui R, Razi B, Aslani S, Imani D, Tabaee SS. The association between Matrix Metallo-proteinases-9 (MMP-9) gene family polymorphisms and risk of Coronary Artery Disease (CAD): a systematic review and meta-analysis. BMC Cardiovascular Disord. 2020;20:1–15. 10.1186/s12872-020-01510-4. PMC7236475 32429880

[14] Dobiásová M. Atherogenic impact of lecithin-cholesterol acyltransferase and its relation to cholesterol esterification rate in HDL (FERHDL) and AIP [log(TG/HDL-C)] biomarkers: the butterfly effect? Physiol Res. 2017;66:193–203. 10.33549/physiolres.933621 28471688

[15] Han SH, Nicholls SJ, Sakuma I, Zhao D, Koh KK. Hypertriglyceridemia and cardiovascular diseases: revisited. Korean Circul J. 2016;46:135–44. 10.4070/KCJ.2016.46.2.135. PMC4805556 27014342

[16] Miller M, Kwiterovich PO. Isolated low HDL-cholesterol as an important risk factor for coronary heart disease. Eur Heart J. 1990;11:9–14. 10.1161/01.ATV.17.1.107. 2073916

[17] James SR, Ray L, Ravichandran K, Nanda SK. High atherogenic index of plasma in subclinical hypothyroidism: implications in assessment of cardiovascular disease risk. Indian J Endocrinol Metab. 2016;20:656–61. 10.4103/2230-8210.190550. PMC5040046 27730076

[18] Tagoe EA, Dwamena-Akoto E, Nsaful J, Aikins AR, Clegg-Lamptey JN, Quaye O. High atherogenic index of plasma and cardiovascular risk factors among Ghanaian breast cancer patients. Exp Biol Med (Maywood). 2020;245:1648–55. 10.1177/1535370220940992. PMC7802380 32640892

[19] Onen S, Taymur I. Evidence for the atherogenic index of plasma as a potential biomarker for cardiovascular disease in schizophrenia. J Psychopharmacol. 2021;35:1120–6. 10.1177/02698811211026450. 34176366

[20] Wang L, Chen F, Xiaoqi C, Yujun C, Zijie L. Atherogenic index of plasma is an independent risk factor for coronary artery disease and a higher SYNTAX score. Angiology. 2021;72:181–6. 10.1177/0003319720949804. 32815391

[21] Al Shawaf E, Al-Ozairi E, Al-Asfar F, Mohammad A, Al-Beloushi S, Devarajan S, et al. Atherogenic index of plasma (AIP) a tool to assess changes in cardiovascular disease risk post laparoscopic sleeve gastrectomy. J Diabetes Res. 2020;2020:1–9. 10.1155/2020/2091341. PMC7422485 32832558

[22] Guo Q, Zhou S, Feng X, Yang J, Qiao J, Zhao Y, et al. The sensibility of the new blood lipid indicator—atherogenic index of plasma (AIP) in menopausal women with coronary artery disease. Lipids in Health and Disease. 2020;19:1–8. 10.1186/S12944-020-01208-8. PMC7041294 32093690

[23] Liberati A, Altman DG, Tetzlaff J, Mulrow C, Gøtzsche PC, Ioannidis JPA, et al. The PRISMA statement for reporting systematic reviews and meta-analyses of studies that evaluate healthcare interventions: explanation and elaboration. BMJ. 2009;339:b2700. 10.1136/BMJ.B2700. PMC2714672 19622552

[24] Wells G, Shea B, O’Connell D, Peterson J, Welch V, Losos M, et al. The Newcastle-Ottawa Scale (NOS) for assessing the quality of nonrandomised studies in meta-analyses. 2014. http://www.ohri.ca/programs/clinical_epidemiology/oxford.asp.

[25] Zhang J, Yu KF. What’s the relative risk? A method of correcting the odds ratio in cohort studies of common outcomes. JAMA. 1998;280:1690–1. 10.1001/JAMA.280.19.1690. 9832001

[26] Shor E, Roelfs D, Vang ZM. The “Hispanic mortality paradox” revisited: meta-analysis and meta-regression of life-course differentials in Latin American and Caribbean immigrants’ mortality. Soc Sci Med. 2017;186:20–33. 10.1016/J.SOCSCIMED.2017.05.049. 28577458

[27] Hozo SP, Djulbegovic B, Hozo I. Estimating the mean and variance from the median, range, and the size of a sample. BMC Med Res Methodol. 2005;5(1):1–10. 10.1186/1471-2288-5-13. PMC1097734 15840177

[28] Onat A, Can G, Kaya H, Hergenç G. “Atherogenic index of plasma” (log10 triglyceride/high-density lipoprotein−cholesterol) predicts high blood pressure, diabetes, and vascular events. J Clin Lipidol. 2010;4:89–98. 10.1016/J.JACL.2010.02.005. 21122635

[29] Al-Shaer MH, Elzaky MM, Farag ESM, Saad MOM. In type 2 diabetes mellitus patients, the atherogenic index of plasma as a marker of coronary artery diseases. Indian J Clin Cardiol. 2021;1:263246362110313. 10.1177/26324636211031362.

[30] Çelik E, Çora AR, Karadem KB. The effect of untraditional lipid parameters in the development of coronary artery disease: Atherogenic index of plasma, atherogenic coefficient and lipoprotein combined index. J Saudi Heart Assoc. 2021;33:244–50. 10.37616/2212-5043.1266. PMC8480409 34631402

[31] Won KB, Jang MH, Park EJ, Park HB, Heo R, Han D, et al. Atherogenic index of plasma and the risk of advanced subclinical coronary artery disease beyond traditional risk factors: an observational cohort study. Clin Cardiol. 2020;43:1398–404. 10.1002/CLC.23450. PMC7724231 32815171

[32] Guo Q, Zhou S, Feng X, Yang J, Qiao J, Zhao Y, et al. The sensibility of the new blood lipid indicator-atherogenic index of plasma (AIP) in menopausal women with coronary artery disease. Lipids Health and Disease. 2020:19;1–8. 10.1186/2Fs12944-020-01208-8. PMC7041294 32093690

[33] Wu TT, Gao Y, Zheng YY, Ma YT, Xie X. Atherogenic index of plasma (AIP): a novel predictive indicator for the coronary artery disease in postmenopausal women. Lipids Health Dis. 2018;17:1–7. 10.1186/S12944-018-0828-Z. PMC6106932 30134981

[34] Cai G, Shi G, Xue S, Lu W. The atherogenic index of plasma is a strong and independent predictor for coronary artery disease in the Chinese Han population. Med (U S). 2017;96:1–6. 10.1097/MD.0000000000008058. PMC5604669 28906400

[35] Shanker J, Kakkar VV. Contribution of classical and emerging risk factors to coronary artery disease in Asian Indians. Int J Cardiol. 2016;214:97–106. 10.1016/J.IJCARD.2016.03.012. 27060267

[36] Priídavkovaá D, Kantárová D, Lišková R, Červeň P, Kovář F, Mokáň M. The role of epicardial fat and obesity parameters in the prediction of coronary heart disease. Vnitr Lek. 2016;62:256–62. 27250602

[37] Dai W, Li Y, Zheng H. Estradiol/testosterone imbalance: impact on coronary heart disease risk factors in postmenopausal women. Cardiology. 2012;121:249–54. 10.1159/000337274. 22572464

[38] Zhou K, Qin Z, Tian J, Cui K, Yan Y, Lyu S. The atherogenic index of plasma: a powerful and reliable predictor for coronary artery disease in patients with type 2 diabetes. Angiology. 2021;72:934–41. 10.1177/00033197211012129. 33949211

[39] Cai G, Liu W, Lv S, Wang X, Guo Y, Yan Z, et al. Gender-specific associations between atherogenic index of plasma and the presence and severity of acute coronary syndrome in very young adults: a hospital-based observational study. Lipids Health Dis. 2019;18:1–9. 10.1186/s12944-019-1043-2. PMC6466804 30987629

[40] Sedhai YR, Basnyat S, Konda P, Koirala A, Prasai P, Raza M, et al. Atherogenic index of plasma is a marker of plaque vulnerability in coronary artery disease. Chest. 2018;154:85A–6A. 10.1016/J.CHEST.2018.08.077.

[41] Xie F, Zhou H, Wang Y. Atherogenic index of plasma is a novel and strong predictor associated with fatty liver: a cross-sectional study in the Chinese Han population. Lipids Health Dis. 2019;18:1–6. 10.1186/s12944-019-1112-6. PMC6739922 31511022

[42] Hernández JL, Baldeón C, López-Sundh AE, Ocejo-Vinyals JG, Blanco R, González-López MA. Atherogenic index of plasma is associated with the severity of hidradenitis suppurativa: a case-control study. Lipids Health Dis. 2020;19:1–6. 10.1186/S12944-020-01377-6. PMC7456502 32861241

[43] Nie X, Gao L, Wang L, Wang J. Atherogenic index of plasma: a potential biomarker for clinical diagnosis of diabetic foot osteomyelitis. Surg Infect (Larchmt). 2020;21:9–14. 10.1089/SUR.2019.020. 31369351

[44] Shen SW, Lu Y, Li F, Yang CJ, Feng YB, Li HW, et al. Atherogenic index of plasma is an effective index for estimating abdominal obesity. Lipids Health Dis. 2018;17:1–6. 10.1186/S12944-018-0656-1. PMC5769292 29334966

[45] Zhu XW, Deng FY, Lei SF. Meta-analysis of atherogenic index of plasma and other lipid parameters in relation to risk of type 2 diabetes mellitus. Prim Care Diabetes. 2015;9:60–7. 10.1016/J.PCD.2014.03.007. 24810146

[46] Zhou Y, Shang X. Usefulness of atherogenic index of plasma for estimating reduced eGFR risk: insights from the national health and nutrition examination survey. Postgrad Med. 2021;133:278–85. 10.1080/00325481.2020.1838138. 33054508

[47] Kubong LN, Nya Biapa PC, Chetcha B, Yanou-Njintang N, Moor Ama VJ, Pieme CA. Relationship between higher atherogenic index of plasma and oxidative stress of a group of patients living with sickle cell anemia in Cameroon. Adv Hematol. 2020;2020. 10.1155/2020/9864371. PMC7103039 32256600

[48] Kammar-García A, López-Moreno P, Hernández-Hernández ME, Ortíz-Bueno AM, Martínez-Montaño MLC. Atherogenic index of plasma as a marker of cardiovascular risk factors in Mexicans aged 18 to 22 years. Proc (Bayl Univ Med Cent). 2020;34:22–7. 10.1080/08998280.2020.1799479. PMC7785179 33456139

[49] Garg R, Knox N, Prasad S, Zinzuwadia S, Rech MA. The atherogenic index of plasma is independently associated with symptomatic carotid artery stenosis. J Stroke Cerebrovasc Dis. 2020;29:105351. 10.1016/J.JVS.2010.03.047. PMC8139324 33045624

[50] Sigala F, Kotsinas A, Savari P, Filis K, Markantonis S, Iliodromitis EK, et al. Oxidized LDL in human carotid plaques is related to symptomatic carotid disease and lesion instability. J Vasc Surg. 2010;52:704–13. 10.1016/J.JVS.2010.03.047. 20573470

[51] Fu L, Zhou Y, Sun J, Zhu Z, Xing Z, Zhou S, et al. Atherogenic index of plasma is associated with major adverse cardiovascular events in patients with type 2 diabetes mellitus. Cardiovas Diabetol. 2021;201:1–11. 10.1186/s12933-021-01393-5. PMC8493717 34610830

[52] Süleymanoğlu M, Rencüzoğulları İ, Karabağ Y, Çağdaş M, Yesin M, Gümüşdağ A, et al. The relationship between atherogenic index of plasma and no-reflow in patients with acute ST-segment elevation myocardial infarction who underwent primary percutaneous coronary intervention. Int J Cardiovas Imaging. 2020;36:789–96. 10.1007/S10554-019-01766-8. 31919706

[53] Bo MS, Cheah WL, Lwin S, Moe Nwe T, Win TT, Aung M. Understanding the relationship between atherogenic index of plasma and cardiovascular disease risk factors among staff of an university in Malaysia. J Nutr Metab. 2018;2018:1–6. 10.1155/2018/7027624. PMC6079547 30116641

[54] Choudhary MK, Eräranta A, Koskela J, Tikkakoski AJ, Nevalainen PI, Kähönen M, et al. Atherogenic index of plasma is related to arterial stiffness but not to blood pressure in normotensive and never-treated hypertensive subjects. Blood Press. 2019;28:157–67. 10.1080/08037051.2019.1583060. 30821503

[55] Ikonomidis I, Makavos G, Lekakis J. Arterial stiffness and coronary artery disease. Curr OpCardiology. 2015;30:422–31. 10.1097/HCO.0000000000000179. 26049393

[56] Kaur M, Rahimi R, Razali F, Mohd Noor N, Omar E, Abdul Manaf Z, et al. Association of coronary artery calcium score with calcification and degree of stenosis: an autopsy study. Malaysian J Pathol. 2019;41:177–83. http://www.mjpath.org.my/2019/v41n2/coronary-artery-calcium.pdf. 31427553

[57] Nam JS, Kim MK, Nam JY, Park K, Kang S, Ahn CW, et al. Association between atherogenic index of plasma and coronary artery calcification progression in Korean adults. Lipids Health Dis. 2020;19:1–7. 10.1186/S12944-020-01317-4. PMC7331149 32615982

[58] Won KB, Heo R, Park HB, Lee BK, Lin FY, Hadamitzky M, et al. Atherogenic index of plasma and the risk of rapid progression of coronary atherosclerosis beyond traditional risk factors. Atherosclerosis. 2021;324:46–51. 10.1016/J.ATHEROSCLEROSIS.2021.03.009. 33813155

[59] Won KB, Jang MH, Park EJ, Park HB, Heo R, Han D, et al. Atherogenic index of plasma and the risk of advanced subclinical coronary artery disease beyond traditional risk factors: an observational cohort study. Clin Cardiol. 2020;43:1398–404. 10.1002/CLC.23450. PMC7724231 32815171

[60] Hartopo AB, Arso IA, Setianto BY Low plasma atherogenic index associated with poor prognosis in hospitalized patients with acute myocardial infarction. Indonesian J Intern Med 2016;48:106–13. http://www.inaactamedica.org/archives/2016/27550879.pdf. 27550879

[61] Wu J, Zhou Q, Wei Z, Wei J, Cui M. Atherogenic index of plasma and coronary artery disease in the adult population: a meta-analysis. Front Cardiovas Med. 2021;0:1927. 10.3389/FCVM.2021.817441. PMC8716758 34977202

[62] Huang H, Yu X, Li L, Shi G, Li F, Xiao J, et al. Atherogenic index of plasma is related to coronary atherosclerotic disease in elderly individuals: a cross-sectional study. Lipids Health Dis. 2021;20:1–9. 10.1186/S12944-021-01496-8. PMC8273949 34247637

