# Supplementary material for: Atherogenic index of plasma and coronary artery disease: A systematic review
Source: Open Med (Wars). 2022 Dec 6;17(1):1915–26. doi: 10.1515/med-2022-0590 (PMC9730543; doi:10.1515/med-2022-0590)
Supplement: Supplementary Material [file med-2022-0590-sm.pdf]

Supplementary material

Appendix 1 . Search strategy

PUBMED

#1"Atherogenic index of plasma"[Title/Abstract] OR "AIP"[Title/Abstract] OR "atherogenic index"[Title/Abstract]

#2"coronary artery disease"[MeSH Terms] OR "Coronary Artery Diseases"[Title/Abstract] OR "left main disease*"[Title/Abstract] OR "Coronary Arteriosclerosis"[Title/Abstract] OR "Coronary Atherosclerosis"[Title/Abstract] OR "coronary disease"[MeSH Terms] OR "Coronary Diseases"[Title/Abstract] OR "coronary heart disease*"[Title/Abstract] OR "ischemic heart disease*"[Title/Abstract]

#1 AND #2

SCOPUS

#1 TITLE-ABS-KEY ("Atherogenic index of plasma" OR "AIP" OR "atherogenic index")

#2TITLE-ABS-KEY ("coronary artery disease" OR "coronary artery diseases" OR "left main disease*" OR "coronary arteriosclerosis" OR "coronary atherosclerosis" OR "coronary disease" OR "coronary diseases" OR "coronary heart disease*" OR "ischemic heart disease*")

#1 AND #2

WEB OF SCIENCE

#1ALL=("Atherogenic index of plasma" OR "AIP" OR "atherogenic index")

#2ALL=("coronary artery disease" OR "coronary artery diseases" OR "left main disease*" OR "coronary arteriosclerosis" OR "coronary atherosclerosis" OR "coronary disease" OR "coronary diseases" OR "coronary heart disease*" OR "ischemic heart disease*")

#1 AND #2

OVID MEDLINE

#1 ("Atherogenic index of plasma" or "AIP" or "atherogenic index").ab.

#2("coronary artery disease" or "coronary artery diseases" or "left main disease*" or "coronary arteriosclerosis" or "coronary atherosclerosis" or "coronary disease" or "coronary diseases" or "coronary heart disease*" or "ischemic heart disease*").ab.

#1 AND #2

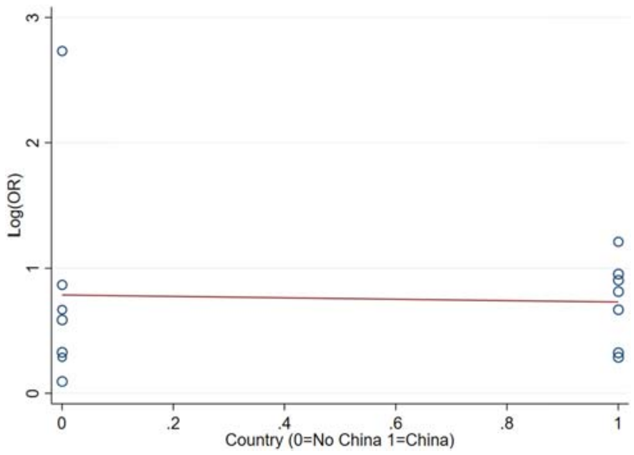

Figure S1: Bubble plot for meta-regression of the study-specific effect sizes and study location.

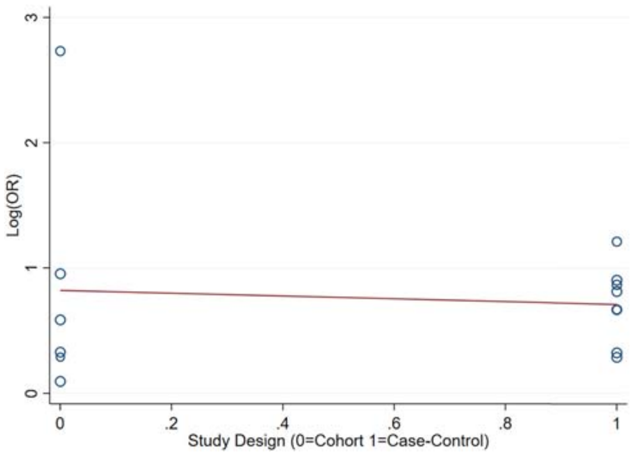

Figure S2: Bubble plot for meta-regression of the study-specific effect sizes and study design.

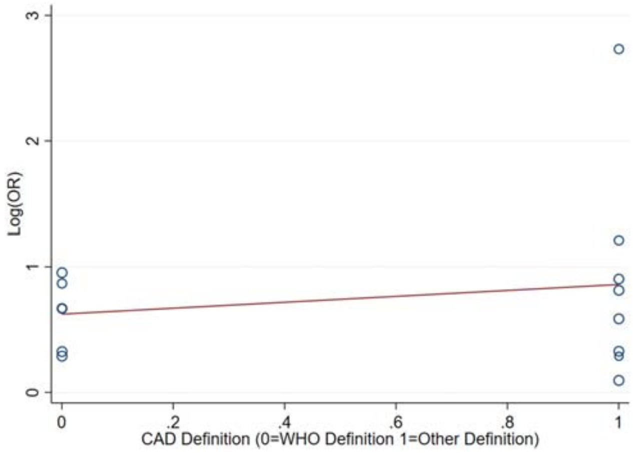

Figure S3: Bubble plot for meta-regression of the study-specific effect sizes and CAD definition.

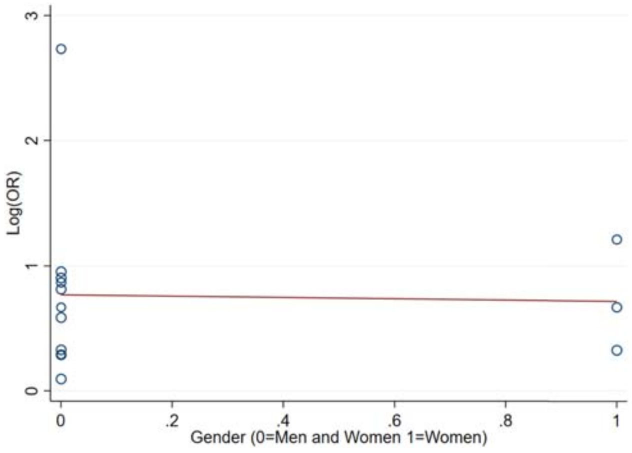

Figure S4: Bubble plot for meta-regression of the study-specific effect sizes and gender.

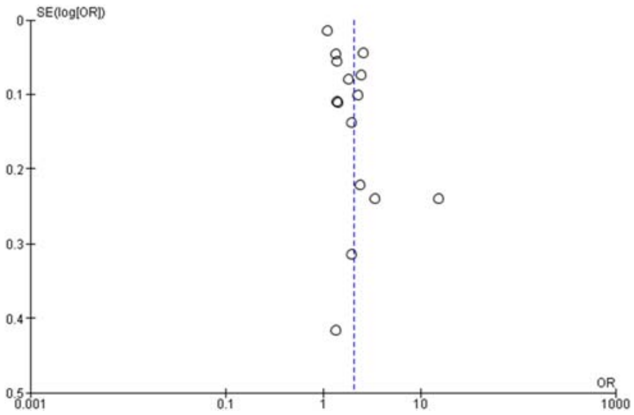

Figure S5: Funnel Plot of the studies that evaluated the association between AIP and risk for CAD.

Table S1: PRISMA Checklist

Section and Topic	Item #	Checklist item	Location where item is reported
**TITLE**			
Title	1	Identify the report as a systematic review.	Title Page
**ABSTRACT**			
Abstract	2	See the PRISMA 2020 for Abstracts checklist.	Title Page
**INTRODUCTION**			
Rationale	3	Describe the rationale for the review in the context of existing knowledge.	Fourth paragraph of the Introduction section
Objectives	4	Provide an explicit statement of the objective(s) or question (s) the review addresses.	Fourth paragraph of the Introduction section
**METHODS**			
Eligibility criteria	5	Specify the inclusion and exclusion criteria for the review and how studies were grouped for the syntheses.	Subheading 2.3
Information sources	6	Specify all databases, registers, websites, organisations, reference lists and other sources searched or consulted to identify studies. Specify the date when each source was last searched or consulted.	Subheading 2.2
Search strategy	7	Present the full search strategies for all databases, registers and websites, including any filters and limits used.	Subheading 2.2 and Supplementary Appendix 1
Selection process	8	Specify the methods used to decide whether a study met the inclusion criteria of the review, including how many reviewers screened each record and each report retrieved, whether they worked independently, and if applicable, details of automation tools used in the process.	Subheading 2.3
Data collection process	9	Specify the methods used to collect data from reports, including how many reviewers collected data from each report, whether they worked independently, any processes for obtaining or confirming data from study investigators, and if applicable, details of automation tools used in the process.	Subheading 2.3
Data items	10a	List and define all outcomes for which data were sought. Specify whether all results that were compatible with each outcome domain in each study were sought (e.g. for all measures, time points, analyses), and if not, the methods used to decide which results to collect.	Subheading 2.5
	10b	List and define all other variables for which data were sought (e.g. participant and intervention characteristics, funding sources). Describe any assumptions made about any missing or unclear information.	Subheading 2.5
Study risk of bias assessment	11	Specify the methods used to assess risk of bias in the included studies, including details of the tool(s) used, how many reviewers assessed each study and whether they	Subheadings 2.4

(*Continued*)

Table S1: *Continued*

Section and Topic	Item #	Checklist item	Location where item is reported
		worked independently, and if applicable, details of automation tools used in the process.	
Effect measures	12	Specify for each outcome the effect measure(s) (e.g. risk ratio, mean difference) used in the synthesis or presentation of results.	Subheading 2.5
Synthesis methods	13a	Describe the processes used to decide which studies were eligible for each synthesis (e.g. tabulating the study intervention characteristics and comparing against the planned groups for each synthesis (item #5)).	Subheading 2.3
	13b	Describe any methods required to prepare the data for presentation or synthesis, such as handling of missing summary statistics, or data conversions.	Subheading 2.5
	13c	Describe any methods used to tabulate or visually display results of individual studies and syntheses.	Subheading 2.5
	13d	Describe any methods used to synthesize results and provide a rationale for the choice(s). If meta-analysis was performed, describe the model(s), method(s) to identify the presence and extent of statistical heterogeneity, and software package (s) used.	Subheading 2.5
	13e	Describe any methods used to explore possible causes of heterogeneity among study results (e.g. subgroup analysis, meta-regression).	Subheading 2.5
	13f	Describe any sensitivity analyses conducted to assess robustness of the synthesized results.	Subheading 2.5
Reporting bias assessment	14	Describe any methods used to assess risk of bias due to missing results in a synthesis (arising from reporting biases).	Subheading 2.4
Certainty assessment	15	Describe any methods used to assess certainty (or confidence) in the body of evidence for an outcome.	Subheading 2.5
**RESULTS**			
Study selection	16a	Describe the results of the search and selection process, from the number of records identified in the search to the number of studies included in the review, ideally using a flow diagram.	Subheading 3.1
	16b	Cite studies that might appear to meet the inclusion criteria, but which were excluded, and explain why they were excluded.	Subheading 3.1
Study characteristics	17	Cite each included study and present its characteristics.	Subheadings 3.1 and 3.2
Risk of bias in studies	18	Present assessments of risk of bias for each included study.	Subheading 3.3
Results of individual studies	19		Subheading 3.4

(*Continued*)

Table S1: *Continued*

Section and Topic	Item #	Checklist item	Location where item is reported
		For all outcomes, present, for each study: (a) summary statistics for each group (where appropriate) and (b) an effect estimate and its precision (e.g. confidence/credible interval), ideally using structured tables or plots.	
Results of syntheses	20a	For each synthesis, briefly summarise the characteristics and risk of bias among contributing studies.	Subheading 3.4
	20b	Present results of all statistical syntheses conducted. If meta-analysis was done, present for each the summary estimate and its precision (e.g. confidence/credible interval) and measures of statistical heterogeneity. If comparing groups, describe the direction of the effect.	Subheading 3.4
	20c	Present results of all investigations of possible causes of heterogeneity among study results.	Subheading 3.4
	20d	Present results of all sensitivity analyses conducted to assess the robustness of the synthesized results.	Subheading 3.4
Reporting biases	21	Present assessments of risk of bias due to missing results (arising from reporting biases) for each synthesis assessed.	Subheading 3.3
Certainty of evidence	22	Present assessments of certainty (or confidence) in the body of evidence for each outcome assessed.	Subheading 3.4
**DISCUSSION**			
Discussion	23a	Provide a general interpretation of the results in the context of other evidence.	Line 1-2 of the Discussion section
	23b	Discuss any limitations of the evidence included in the review.	Paragraph 9 of the Discussion section
	23c	Discuss any limitations of the review processes used.	Paragraph 9 of the Discussion section
	23d	Discuss implications of the results for practice, policy, and future research.	Paragraph 9 of the Discussion section
**OTHER INFORMATION**			
Registration and protocol	24a	Provide registration information for the review, including register name and registration number, or state that the review was not registered.	Subheading 2.1
	24b	Indicate where the review protocol can be accessed, or state that a protocol was not prepared.	Subheading 2.1
	24c	Describe and explain any amendments to information provided at registration or in the protocol.	Subheading 2.1
Support	25	Describe sources of financial or non-financial support for the review, and the role of the funders or sponsors in the review.	AUTHOR'S STATEMENTS
Competing interests	26	Declare any competing interests of review authors.	AUTHOR'S STATEMENTS
	27		AUTHOR'S STATEMENTS

(*Continued*)

Table S1: *Continued*

Section and Topic	Item #	Checklist item	Location where item is reported
Availability of data, code and other materials		Report which of the following are publicly available and where they can be found: template data collection forms; data extracted from included studies; data used for all analyses; analytic code; any other materials used in the review.	

Table S2: CAD definitions for each study

Author	Definition of CAD
Onat A et al.	Diagnosis was determined on the basis of the presence of angina pectoris, of a history of myocardial infarction with or without accompanying Minnesota codes of the electrocardiogram, or by a history of myocardial revascularization.
Raj-Sedhai Y et al.	Acute coronary syndrome based on the indication for cardiac catheterization
Al-Shaer MH et al.	CAD was defined in accordance with the 1979 WHO diagnostic criteria
Celik E et al.	CAD was defined in accordance with the 1979 WHO diagnostic criteria
Wang L et al.	CAD was defined in accordance with the 1979 WHO diagnostic criteria
Won K et al.	Heavy coronary calcification or obstructive coronary plaque
Guo Q et al	CAD was defined in accordance with the 1979 WHO diagnostic criteria
Wu T et al.	CAD was defined in accordance with the 1979 WHO diagnostic criteria
Cai G et al.	CAD was defined in accordance with the 1979 WHO diagnostic criteria
Pridavkova D et al.	Coronary stenosis diagnosed by coronary angiography
Shanker J et al.	CAD was diagnosed by coronary angiography (>70% stenosis in one major epicardial vessel or >50% stenosis in two or more smaller coronary vessels) and electrocardiogram
Dai W et al.	Coronary heart disease: >70% occlusion of at least one major coronary artery
Zhou K et al.	50% diameter stenosis of at least 1 major coronary artery according to angiograms.
Gaojun C et al.	Acute coronary syndrome, including acute myocardial infarction and unstable angina pectoris .

CAD: Coronary artery disease.

Table S3: Newcastle - Ottawa quality assessment scale for included studies

NEWCASTLE - OTTAWA QUALITY ASSESSMENT SCALE FOR COHORT STUDIES

STUDY	SELECTION				COMPARABILITY	OUTCOME				
	Representativeness of the exposed cohort	Selection of the non-exposed cohort	Ascertainment of exposure	Demonstration that outcome of interest was not present at start of study	Comparability of Cohorts on the Basis of the Design or Analysis Maximum : **	Assessment of outcome	Was follow-up long enough for outcomes to occur	Adequacy of follow up of cohorts	SCORE	Evidence quality
Raj-Sedhai Y et al.		*	*	*			*	*	5	Moderate risk of bias
Wang L et al.	*	*	*		**	*	*		7	Low Risk of bias
Won K et al.	*	*	*		*	*		*	6	Low Risk of bias
Pridavkova D et al.		*	*		*	*	*	*	6	Low Risk of bias
Shanker J et al.	*	*	*	*	**	*	*	*	8	Low Risk of bias
Onat A et al.	*		*	*	**	*	*		7	Low Risk of bias

NEWCASTLE - OTTAWA QUALITY ASSESSMENT SCALE FOR CASE-CONTROL STUDIES

STUDY	SELECTION				COMPARABILITY	OUTCOME				
	Is the case definition adequate?	Representativeness of the cases	Selection of Controls	Definition of Controls	Comparability of cases and controls on the basis of the design or analysis (Maximum : **)	Ascertainment of exposure	Same method of ascertainment for cases and controls	Non-Response rate	SCORE	Evidence quality
Al-Shaer HM et al.	*	*					*	*	4	Moderate risk of bias
Cai G et al.	*	*			**	*	*		6	Low Risk of bias
Gaojun C et al.	*	*			**	*	*		6	Low Risk of bias
Celik E et al.	*	*		*		☆	*		5	Moderate risk of bias
Dai W et al.	*	*		*		*	*		5	Moderate risk of bias
Guo Q et al	*	*		*	**	*	*		7	Low Risk of bias
Wu T et al.	*	*		*	**	*	*		7	Low Risk of bias
Zhou K et al.		*		*	**	*	*		6	Low Risk of bias
